# A moment from before 365 Ma frozen in time and space

**DOI:** 10.1038/srep14191

**Published:** 2015-09-18

**Authors:** Błażej Błażejowski, Piotr Gieszcz, Carlton E. Brett, Marcin Binkowski

**Affiliations:** 1 Institute of Paleobiology, Polish Academy of Sciences, Twarda 51/55, 00-818 Warszawa, Poland; 2 Association of Polish Climatologists, Krakowskie Przedmieście 30, 00-927 Warszawa, Poland; 3 Department of Geology, University of Cincinnati, Cincinnati, Ohio, USA, 45221-0013; 4 Department of Computer Science and Materials Science, University of Silesia, Katowice, Poland

## Abstract

This study presents a detailed analysis of an exceptionally well-preserved articulated specimen of the trilobite *Trimerocephalus* from the Late Devonian of the Holy Cross Mountains in Poland. X-ray microtomography reveals the oldest direct evidence for a moulting episode known from the fossil record. The process of moulting as well as associated features observed in the investigated specimen are interpreted by comparison with extinct and extant Xiphosurida arthropods, which survived global P/T extinction and are among the closest extant relatives of trilobites. A very special moment frozen in time and space millions years ago provides rare insights into the behavior and physiology of these long-extinct arthropods.

## Introduction

**Figure 1:**
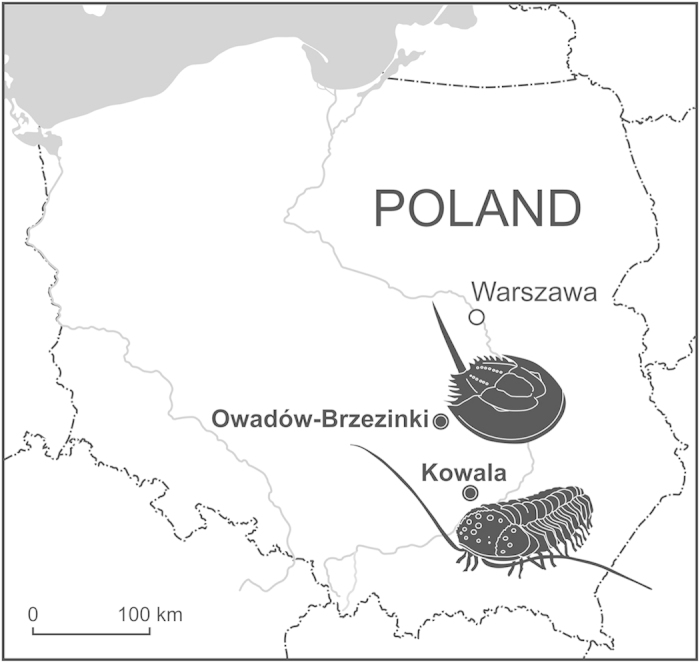
Contour of Poland with the location of Kowala (50° 47′ 46.21” N, 20° 33′ 57.52” E) and Owadów-Brzezinki (51° 22′ 34.53” N, 20° 89′ 07.86” E) quarries. Drawings by B. Błażejowski.

Trilobites, a group of marine arthropods which came to prominence in the ‘Cambrian explosion’, finally disappeared during the end Permian Period mass extinction, about 250 million years ago, after a prolonged decline in diversity and abundance. The trilobites were among the most successful of all the early animals, roaming the oceans for over 270 million years. The recent discovery of a three-dimensionally preserved articulated specimen of the blind trilobite *Trimerocephalus chopini* from the Late Devonian (Early Famennian; ~365 Ma) at the Kowala Quarry, near Kielce, Poland (Fig. 1a,b) adds significantly to our understanding of the subphylum Trilobitomorpha. In order to put these observations in a broader context, we extended our study to another group of arthropods - the xiphosurid horseshoe crabs, commonly regarded as the closest extant relatives of trilobites^1^. For a comparison, we chose an exceptionally well-preserved horseshoe crab *Crenatolimulus* sp. nov. from the latest Jurassic (Late Tithonian) sediments at the Owadów-Brzezinki Quarry, near Tomaszów Mazowiecki in Central Poland (Fig. 1a,c). The aim of this paper is to present evidence about the mode of preservation, behavior and physiological effects of moulting and its relationship to arthropod survivability.

The blind phacopine genus *Trimerocephalus*^2^ has an almost worldwide distribution in the Famennian^3^, except for North America. Numerous recent finds of *Trimerocephalus*, commonly forming a line (or ‘queue’) of a few to more than 20 individuals aligned one after another on the upper surfaces of shale beds, are reported from Kowala Quarry, in the Holy Cross Mountains, South-central Poland^3^,^4^. From this collection, several specimens were chosen for detailed investigation with X-ray microcomputed tomography (XMT). This provides precise information about the shape and dimensions of internal structures preserved in the rock matrix, giving an opportunity to study valuable fossils without a risk of damage during preparation^5^,^6^. Tomographic data after digital processing enable the construction of an isosurface-based and volume-based 3-D model (a ‘virtual fossil’), which can be manipulated, dissected or measured interactively. A sample visualization of the discussed articulated specimen of the *Trimerocephalus chopini* is available at http://www.paleo.pan.pl/people/Blazejowski/SOM_T_chopini_TIME_SPACE.mpg.

**Figure 2:**
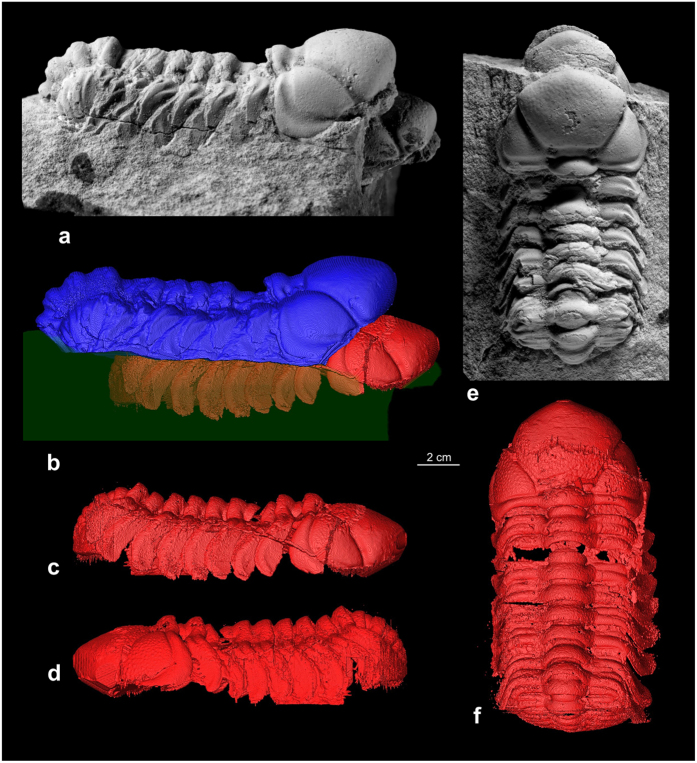
*Trimerocephalus chopini* from Early Famennian marly shales at Kowala Quarry, Holy Cross Mountains, central Poland. (**a**). oblique lateral view (SEM); (**b–d**). oblique lateral view (XMT), (**e**). dorsal view (SEM); (**f**). dorsal view (XMT).

Under X-ray tomography the specimen of *Trimerocephalus chopini* is seen to consist of two adjacent exoskeletons, of which the upper one is a an articulated thorax and pygidium with the cephalon associated but detached and rotated slightly upward. These skeletal remains are interpreted as a moult ensemble (exuvium) preserved *in situ* shortly after separating from the moulting individual recognizable as the lower articulated exoskeleton (Fig. 2a,b). The latter appears to reflect the “soft-shelled” individual that died exactly after time of moulting. The increased number of segments visible in the “soft-shelled” trilobite after moulting illustrates the process of growth typical of all arthropods (Fig. 2b,c). A new segment is developed under the old, loosening exoskeleton, and until the moult is loosened, it remains compressed, limited by the total volume of the old exoskeleton (Fig. 2b,c,f). As in extant arthrtopods the individual inflates to reach new dimensions directly after moulting.

The preservation of this extraordinary composite moult and carcass of *Trimerocephalus* raises intriguing issues of taphonomy and provides further insights into trilobite moult behavior. The articulated preservation of both the carcass and the exuviated exoskeleton requires not only mortality of this individual immediately following moulting but also synchronous entombment of both skeletons within the sediment. This could have happened as a result of rapid, live burial but, in that case, the carcass exoskeleton and exuvium would probably have remained pancaked rather than separated by a layer of sediment. A more plausible scenario was suggested by newly documented specimens of the Silurian–Early Devonian phacopid trilobite *Paciphacops*^7^ from Argentina^8^. In this case, specimens from concretions show the cephalon to be slightly disarticulated and rotated upward from the thoracopygidium^8^. Rustan *et al.* surmised that these trilobites moulted while burrowed shallowly within the sediment. Similar specimens of *Phacops* from the Lower Devonian of Morocco show the cephalon detached and “floating” above the articulated thoracopygidium^9^. In all of these cases, the “soft shelled” trilobite was not apparently preserved, presumably because the animals crawled out of the sediment following successful moulting, leaving the moult parts suspended within the mud. In the illustrated *Trimerocephalus* specimen, the upper, moulted exoskeleton is quite similar in configuration to the other exoskeletons that were inferred to have been left within the sediment. In this specimen, however, the moulting was evidently not successful and the animal died within the sediment shortly after extricating itself from the old exoskeleton. We infer that *Trimerocephalus* also moulted within the sediment and, in this case, left its moult slightly before it also died within the sediment. It is notable that the inferred soft-shelled carcass is preserved below and somewhat forward of the exuvium as might be expected of an animal that had extricated itself from its old exoskeleton (Fig. 2). The cause of mortality is, of course, not known but might be due to stress of moulting or possibly, given the context, death by hypoxia or hydrogen sulfide toxicity within the sediment.

It is also pertinent to enquire how the newly moulted “soft-shelled” trilobite could theoretically be preserved at all. However, it is evident that some individuals did have enough mineralization following moulting to be preserved. Speyer and Brett^10^ illustrated a specimen of a very thin, wrinkled exoskeleton of *Eldredgeops rana* found adjacent to other individuals with typical thick, dark calcitic exoskeletons; a few specimens of this sort have been noted and interpreted as carcasses of soft shelled individuals. This is in line with the wrinkling observed here. It is also possible that this was a case of delayed moulting so that the new exoskeleton was more strongly developed than in most “soft shelled” trilobites; hence, its strongly defined preservation.

### Wrinkles and scars

**Figure 3:**
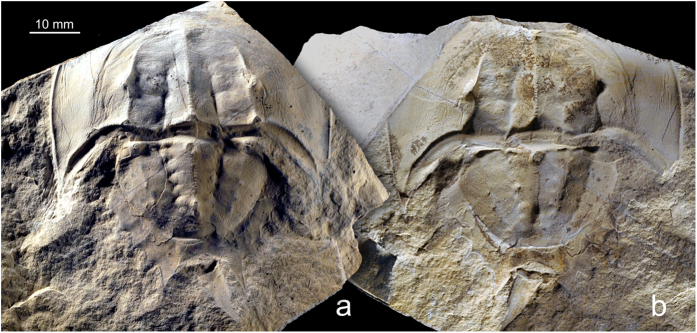
Three-dimensionally preserved representatives of Late Jurassic horseshoe crabs from Owadów-Brzezinki, Poland. *Crenatolimulus* sp. nov. (ZPAL X.1/O-B/14.1). (**a**) Negative and (**b**) positive (rock slab with imprint).

Observed features of the “soft-shelled” trilobite specimen, such as noticeable compression and the presence of wrinkles in the area of the anterior cephalon (Fig. 2e) clearly indicate that the death of this individual occurred just after the process of moulting. Moulting is a common feature of all arthropods including extant and extinct horseshoe crabs, therefore we searched for analogies among an exceptionally well-preserved collection of Late Jurassic horseshoe crabs^11^,^12^. These are among the rarest of macrofossils and it is noteworthy that the collection consists of only very well preserved groups of juvenile specimens found^13^. In the latter case, we observed very similar wrinkles in many (but not all) investigated horseshoe crab specimens (Fig. 3). The wrinkling along the anterior prosoma has been interpreted previously as an effect of sediment compaction^14^, and this can be the reason in many cases of partly or entirely flattened specimens, but the specimen presented here is very well three-dimensionally preserved (Fig. 3). Taking into consideration the above mentioned course of moulting, another explanation is suggested: when an individual dies during or immediately following moulting, “decompression” does not occur (or occurs only to a limited extent) and results in a form of ‘wrinkles’ that can be observed in the fossillised state. Such wrinkling may be especially well preserved when, as in the case of the *Trimerocephalus* specimen, the animal died within the sediment. Similar features were observed in laboratory environment, where a 5–6 year old juvenile of modern Atlantic horseshoe crab (*Limulus polyphemus*) died in a difficult moulting episode, which resulted in deformation in limbs and wrinkles on its dorsal exoskeleton (Y. Iwasaki, pers. obs.). Possibly the cause of death in all these cases is the same: the effort and energetic loss during the moulting could be so excessive under degraded environmental conditions, that many weak juveniles simply cannot survive the event. This neatly explains only juvenile specimens are commonly find in the fossil record.

The preservation of many specimens very close to each other may be the effect of behavioral phenomena observed in recent horseshoe crabs. Young individuals spend their first years rapidly increasing in size, prior to venturing out into deeper waters, in the so called “nursery grounds^15^,^16^”. By analogy, this may indicate that the depositional area of fossil horseshoe crabs could have acted as a nursery ground for numerous individuals, which moulted under somewhat adverse conditions associated with their rapid burial and did not survive the process, forming concentrations of fossils. This may be the one of the reasons why we often observe concentrations of juvenile fossil arthropods. The lack of preserved adult individuals (known only from trackways^17^,^18^,^19^) in all collections of Jurassic horseshoe crabs is a strong premise supporting that assumption.

A somewhat similar scenario could apply in the case of the Late Devonian *Trimerocephalus*. As noted, this specimen is associated with ‘queues’ of conspecific trilobites, which may represent rows of trilobites that migrated into certain areas, possibly deep offshore setting to breed. An abundance of minute individuals (meraspids?) in association with the rows may represent recently metamorphosed juveniles growing in a “nusery ground”. It is possible, that in a restricted setting, low oxygen and/or high hydrogen sulfide may have been more detrimental to mature trilobites, many of which, including the specimen described herein, appear to have expired literally in their tracks.

## Additional Information

**How to cite this article**: Błażejowski, B. *et al.* A moment from before 365 Ma frozen in time and space. *Sci. Rep.* **5**, 14191; doi: 10.1038/srep14191 (2015).
